# Development of computations in bioscience and bioinformatics and its application: review of the Symposium of Computations in Bioinformatics and Bioscience (SCBB06)

**DOI:** 10.1186/1471-2105-7-S4-S1

**Published:** 2006-12-12

**Authors:** Youping Deng, Jun Ni, Chaoyang Zhang

**Affiliations:** 1 Department of Biological Sciences, University of SouthernMississippi, Hattiesburg, MS 39406, USA; 2 Department of Computer Science, the University of Iowa, Iowa City, IA 52242, USA; 3 School of Computing, University of Southern Mississippi,Hattiesburg, MS 39406, USA

## Abstract

The first symposium of computations in bioinformatics and bioscience (SCBB06) was held in Hangzhou, China on June 21–22, 2006. Twenty-six peer-reviewed papers were selected for publication in this special issue of BMC Bioinformatics. These papers cover a broad range of topics including bioinformatics theories, algorithms, applications and tool development. The main technical topics contain gene expression analysis, sequence analysis, genome analysis, phylogenetic analysis, gene function prediction, molecular interaction and system biology, genetics and population study, immune strategy, protein structure prediction and proteomics.

## Introduction

This BMC Bioinformatics supplement consists of 26 papers peer-reviewed and selected from the First Symposium of Computational Biology and Bioinformatics (SCBB) in conjunction with the International Multi-Symposiums on Computer and Computational Sciences (IMSCCS|06) held in Zhejiang University, Hangzhou, China on June 21–22, 2006. A total of 230 conference participants, including research scientists, faculty, and graduate students with different disciplines and backgrounds in both academia and industry, attended the conference. This symposium was designed to report the progress of bioinformatics and computational biology from the recent work presented by the authors, as well as to bring together computational biology and bioinformatics researchers to discuss fundamental methods, algorithms, and research software for analyzing biological data; thus, to establish future collaborations. The authors, coming from 7 countries and 36 research institutions all over the world, contributed their work to this special issue.

## Process of submission and reviews

We received submissions both from the presenters at the symposium and from non-presenters. Submitted manuscripts were intensively reviewed by at least two referees. The quality of each paper was evaluated based on the contribution to computational biology and bioinformatics. Technical novelty and expression rigor in the methodology was required. The accepted papers in the specific issue covered a broad range of subject areas and can be mainly divided into the following categories:

### Gene expression analysis

Most papers regarding gene expression analysis focused on microarray data analysis. 9 papers in the proceedings mainly address micorarray experiments, data analysis and tool development, which indicates that microarray data analysis is still the hottest topic in bioinformatics and computational biology. Perkins et al [1] presented their work of comparing the gene expression difference between primary heptocyte cell culture and liver tissue after exposure to hexahydro-1, 3, 5-trinitro-1, 3, 5-triazine, which is a toxic chemical that contaminates soil and ground water, which affects human and animal health. The microarray data analysis was performed using JMP Genomics from SAS Institute Inc. (Cary, NC. ) and ArrayTrack () and EASE [2]. They found that the absolute common differentially expressed gene list between primary heptocyte cell culture and liver tissue is not so high, but if the KEGG pathway functional category is considered, the overlapped common functional gene list is much greater. Their results suggest that we need to take care of the extrapolating effects from *in** vitro* and *in** vivo* models.

Because microarray experiments are expensive, it is important to determine an appropriate sample number for a micorarray experiment. Wu and his colleagues [3] have developed a method to determine the minimum microarray samples such as the minimum time points for the micorarray researchers. Their basic idea is to use hierarchical clustering to obtain the gene expression patterns in a microarray experiment. Using time series data as examples, they found that gene expression patterns could be "saturated" at a certain time point, and more time points will not furhter contribute to pattern discrimination so they are unnecessary.

Two presenters focused on the methods of identifying differentially-expressed genes. Yang et al [4] investigated the effect of sample imbalance on differentially-expressed gene identification. When we try to find the differentiated gene list between two conditions such as control and treatment, it often happens that the sample number of two conditions might not be equal. Under these circumstances, choosing a right method to identify differentially-expressed genes is critical. By using two evaluation models, they compared 6 popular methods to select differentially expressed genes in two real datasets and one simulated data, and found that different methods turned out different results in these unbalanced data. So they suggested that care should be taken in choosing the right method for identifying-differentially expressed genes on specific unbalanced data. Based on their fuzzy set theory, Liang et al [5] developed a new approach called fuzzy membership test (FM-test) to identify differentially-expressed genes. They assign FM- d-value to the genes that can distinguish two conditions. They applied their methods to both diabetes and lung cancer microarray data and found some existing genes for diabetes and lung cancer, as well as some new genes related to diabetes or lung cancer, indicating that their method is effective.

There were two papers that concentrated on the validation of clustering algorithms for gene expression data. Yin et al. [6] compared the runtime performance of three major clustering algorithms: Hierarchical Clustering, Self-Organizing Map (SOM) and Self Organizing Tree Algorithm (SOTA) using yeast microarray dataset, they found that SOTA is the most efficient algorithm, followed by SOM, and Hierarchical algorithm is the slowest one. They recommend using SOTA because it has the advantages of both SOM and hierarchical clustering algorithms. Meanwhile, they presented a novel data mining tool, called "Cluster Diff", for similarity analysis of clusters. Datta and Datta [7] compared six popular clustering algorithms including UPGMA, K-Means, Diana, Fanny, Model-Based and SOM using both SAGE and cDNA microarray data. Judging by both the statistical and the biological functional consistency of the clusters, they found that overall UPGMA is a good performer, but they thought the absolute winner may not be clear in the resulting data. They suggested that choosing a right clustering algorithm for specific dataset and their validation method is very promising.

A recent report [8] showed that the Support Vector Machine (SVM) algorithm runs better than many other classification algorithms, but it is extremely time-consuming for analyzing large microarray datasets. Zhang et al [9] report an innovative tool called Parallel Multicategory Support Vector Machine (PMC-SVM) based on the sequential minimum optimization-type decomposition method for support vector machine (SMO-SVM). Tested on four large microarray datasets, they found that PMC-SVM was found to drastically improve the performing efficiency without losing any accuracy, in contrast to the serial SVM algorithm.

Guoqing Lu et al [10] developed a data mining tool called AffyMiner, for specifically mining Affymetrix microarray data. It can be used to identify differentially-expressed genes, perform clustering, and classify interesting gene function according to Gene Ontology (GO) and pathway analysis. Classifying microarray data (such as a cancer microarray data to distinguish multiple classes corresponding to different subtypes of a specific cancer) is important. It can be used for disease diagnosis and prognosis. Since SVM is a very good algorithm for microarray data classification, it is very useful to create a graphical user interface (GUI) for use of SVM for data analysis. Pirooznia and Deng [11] have developed a user-friendly Java GUI application allowing users to perform SVM training, classification and prediction. They demonstrated that their software can accurately classify genes into functional categories based upon expression data from DNA microarray experiments. The software provides various kernel functions for users to choose the best way for classifying their data, and it is freely available at .

## Sequence analysis

### Shortest common supersequences (SCS)

There were five papers studying sequence analysis. As an important transcription binding site, TATA is an old topic. However, Shi and Zhou [12] made some new findings on the frequency distribution of TATA Box and its extension sequences on human promoters. Based on their extensive statistical analysis, they divided 16 TATA elements into 3 distribution patterns. Interestingly, they found that 14 TATA extension sequences were new TATA Box elements. Mao and Zheng [13] proposed a new approach to find common human transcription factor binding motifs in the upstream regions of co-regulated genes resulted from gene expression experiments. They employed comparative genomics as well as *de novo *motif finding strategy to identify common motifs. The method turned out to be better than existing methods. Ning and Leong [28] reported a novel heuristic algorithm, the Deposition and Reduction algorithm, for detecting the shortest common supersequences (SCS). They prove that their algorithm runs better than or is comparative to the existing popular used algorithms, especially when more long sequences are used to locate SCS.

### Longest common sequence (LCS)

Computation for finding the longest common sequence (LCS) of multiple biosequences is the fundamental task and challenge due to intensive computation. To speedup the computation has significance in bioinformatics. Chen et al [26] developed a parallel algorithm for finding LCS. In their algorithm, an effective pruning technique is deployed which can significantly reduce the computational complexity. The algorithm is implemented using a message passing interface, a parallel library to parallelize the program. The experimental results on gene sequences in the *tigr *database show the parallel algorithm is optimal and highly efficienct.

Two papers dealt with protein sequence families. Chen et al [15] proposed a new method to cluster protein sequences. They named this new method as SEQOPTICS (sequence clustering with OPTICS), which is based on the approach OPTICS (Ordering Points to Identifying the Clustering Structure). They demonstrated that their method performs better than well known existing methods. Hydrophobin proteins are fungal proteins that have been used to make paints. Yang et al [16] define common new motif patterns for hydrophobin protein family. Based on the newly identified patterns and the existing pattern of the protein family, they find 9 new hydrophobin proteins that have not yet been named as hydrophobins, which provides new sources for potential industrial applications.

## Biological function analysis

It is important to understand the functionalities of biological structures. Such efforts can be transformed to a process of finding a maximum common subgraph (MCS) graphically between two different biological structures. In this domain people utilize parameterized computation in the MCS study. Huang et al [27] derived a new lower bound for the exact algorithms of the maximum common induced subgraph. The authors also investigated the upper bound effects.

## Genome analysis

Lu et al [17] developed a user friendly web tool, GenomeBlast to compare small genomes. This tool can be used to identify homologous and unique genes among compared genomes, as well as to view genome distribution graphically and construct genome phylogenetic trees. The web server is available to any users.

## Phylogenetic analysis

Based on the ant-colony algorithm, Qin et al [18] propose an innovative approach to construct phylogenetic tree. This is a distanced based method. They improved the ant-colony algorithm by developing an adaptive heuristic clustering algorithm. They demonstrate that their adaptive algorithm is better than Genetic Algorithm (GA) for constructing phylogenetic tree. This new method provides an alternative approach for finding DNA, protein relationship based on phylogenectic tree.

## Gene function prediction

Although the genomes of many organisms have been sequenced, the gene function is largely unknown. Li et al [19] presented a new approach, Fuzzy Nearest Clusters to predict the function of unclassified genes based on microarray data. They assume that the genes in the same cluster or subgroup should have similar functions, and assign the function of the unclassified genes to the function of genes whose function is known in the same cluster of subgroup. Because they use the fuzzy strategy, the approach holds an advantage that it can predict multiple roles for an unclassified gene.

Duan et al [20] surveyed the relationship between protein sequence similarity and their Gene Ontology (GO) function terms. They found that protein sequences that have GO functions tend to have sequence similarity. But they also suggested more evidence should be considered to accurately predict a gene function besides sequence similarity.

Stepanova et al [25] developed a method which can be used for the prediction of hormone-response elements (HRE) de novo. This method can handle large groups of transcription factor binding sites. Their model has been proved by their experimental results.

## Molecular interaction and system biology

Azuma et al [27] focused on molecular-level dynamics to affect molecular properties at the cellular level. Based on a particle model, they designed an algorithm to simulate the chemical reaction-diffusion dynamics of molecules. They evaluated their simulation algorithm in a reversible enzyme reaction model and demonstrated its efficiency. This algorithm provides a quantitative way to model the molecule interaction dynamics and it is very useful for understanding the mechanism of molecular interaction as well as cellular signaling and metabolism.

## Genetics and population study

Zhang et al [22] proposed a two-stage approach to identify haplotype frequencies in pedigrees. The two stages include the haplotyping stage and the estimation stage. They demonstrated that their new method performs faster and more accurately than other existing well known software.

## Structure prediction

The Chou-Fasman's method is a famous method to predict protein secondary structure. Hang Chen et al [23] proposed a new version of Chou-Fasman's method by significantly improving its performance from three aspects, which include changing the values in the nuclear regions, using new secondary structure parameters and modifying Chou-Fasman rules. It turns out that their improved method performs much better than the original Chou-Fasman's method and is comparable to other well known methods.

## Biological immune system

For the immune system of an organism, there exits artificial intelligent technology such as dealing with immune selection, memory storage, immune metabolism, and density control. Qin et al [24] proposed an adaptive ant colony algorithm that simulates the behavior of biological immune system. The solutions to NP-hard problems are much more diversified, so that the stagnation and premature phenomena in such biological system can be avoided.

## Future Meetings

The Symposium of Computations in Bioinformatics and Bioscience is an annual conference. The second symposium is scheduled to be held in the United States of America. The updated information about the next SCBB06 symposium can be found at Web site: .
